# Stimulation Strategies for Tinnitus Suppression in a Neuron Model

**DOI:** 10.1155/2018/5215723

**Published:** 2018-07-30

**Authors:** Alessandra Paffi, Francesca Camera, Chiara Carocci, Francesca Apollonio, Micaela Liberti

**Affiliations:** Sapienza University of Rome, Via Eudossiana 18, 00184 Rome, Italy

## Abstract

Tinnitus is a debilitating perception of sound in the absence of external auditory stimuli. It may have either a central or a peripheral origin in the cochlea. Experimental studies evidenced that an electrical stimulation of peripheral auditory fibers may alleviate symptoms but the underlying mechanisms are still unknown. In this work, a stochastic neuron model is used, that mimics an auditory fiber affected by tinnitus, to check the effects, in terms of firing reduction, of different kinds of electric stimulations, i.e., continuous wave signals and white Gaussian noise. Results show that both white Gaussian noise and continuous waves at tens of kHz induce a neuronal firing reduction; however, for the same amplitude of fluctuations, Gaussian noise is more efficient than continuous waves. When contemporary applied, signal and noise exhibit a cooperative effect in retrieving neuronal firing to physiological values. These results are a proof of concept that a combination of signal and noise could be delivered through cochlear prosthesis for tinnitus suppression.

## 1. Introduction

Tinnitus is a debilitating perception of sound in the absence of external auditory stimuli that affects more than 10% of the world population [1–3] and tends to increase with the age [2, 3].

The origin of this debilitating disorder may be central or peripheral; i.e., it can originate in the cochlea, in the primary hearing cortex or in any other point of the auditory pathway [4].

Based on frequency and permanence of sound perception, tinnitus is classified in continuous low frequency tinnitus (CLFT) for frequencies below 100 Hz, continuous high frequency tinnitus (CHFT) for frequencies above 3 kHz, and transient spontaneous tinnitus (TST) [5]. Several studies [6, 7] confirm that the CHFT is the most widespread tinnitus typology, generally associated with a reduction of cochlear functionality at high frequency, due to a damage of the basal section of the cochlea. In the tonotopic organization of sound perception [8], the cochlea basal section encodes for high frequency stimuli, above 3 kHz.

This close association between tinnitus and hearing loss suggests that, in many cases, it is due to an impairment of the outer hair cells (OHC) of the cochlear basal section that, in turn, induces a pathologic state of depolarization of the inner hair cells (IHC) [9].

In 1995 Le Page [9] proposed a cochlear model to explain tinnitus origin. The OHCs determine the hair deflection of the IHCs that, in turn, depolarize the acoustic fibers. In physiologic conditions, in the absence of an external stimulus, the OHCs fix the operating point on the IHC transfer function (acoustic neuron depolarization versus IHC hair deflection) to a position that brain recognizes as absence of sound. When the OHCs are damaged, the control input to the IHCs gets lost with a consequent shift of the operating IHC point and a permanent firing rate of the acoustic fiber interpreted by the brain as a real acoustic pattern [9].

This modification of the nerve fiber firing pattern due to OHC impairment was experimentally observed in different animal models [10–13].

Several experimental studies [14–16] revealed that an electric stimulation of the cochlea, delivered through cochlear prosthesis or transtympanic electrode, could alleviate tinnitus perception in a significant percentage of treated patients. McKerrow and colleagues [14] used continuous wave (CW) high frequency signals (2-6 MHz) superposed to a Gaussian white noise (GWN), whereas other authors used pulse trains with repetition frequency up to 5 kHz [15, 17]. Recently, Tyler and colleagues [18] efficiently used pulsed modulated signals delivered to the Vagus nerve on human volunteers.

However, the electric signals delivered in stimulation, in terms of type (CW, pulse train, white noise), frequency content, amplitude, and modulation, were empirically chosen and their mechanisms of action on the auditory fibers were not defined.

Moving from a recent study by the authors [19] showing an inhibitory effect of an electric exogenous stimulation on a hyperexcited neuronal network model, it was hypothesized that an electric stimulation may interfere with the neuron firing pattern of a pathologically polarized acoustic neuron by reducing its firing rate to the physiologic one.

Aim of this work is to verify such a hypothesis and to study the efficacy of a combination of signal and noise in tinnitus inhibition, using a simple model of a hyperexcited auditory fiber.

In a biomedical perspective, the final aim is to deliver this stimulation to the auditory nerve using cochlear prosthesis to suppress tinnitus in patients with acoustic impairment.

## 2. Models and Methods

### 2.1. Neuron Model

To describe the single Ranvier node of an auditory fiber, a stochastic Hodgkin-Huxley (HH) model was used [20–22]. In this model, the neuronal membrane patch is represented by an electrical equivalent, in which the balance of the currents per unit area is given by

(1) CmdVdt=−glV−El−gKV−EK−gNaV−ENa+I0

where *C*_*m*_ is the unit area capacitance that takes into account the dielectric properties of the membrane phospholipidic bilayer, *V* is the transmembrane potential,* g*_*Na*_,* g*_*K*_,* g*_*l*_ are sodium, potassium and leakage conductances per unit area, respectively, and* E*_*Na*_,* E*_*K*_,* E*_*l*_ are the reversal potentials of the corresponding current densities. Finally,* I*_0_ is the bias current density that controls the transition between the resting state and the firing activity of the neuron [23]. For the deterministic HH model at 6.3°C, the threshold value above which the neuron starts its firing activity is equal to 6.3 *μ*A/cm^2^ [23].

Despite the model limitation concerning the operating temperature equal to 6.3°C, it is simple, very well characterized in terms of neuronal response as a function of model parameters, and the most used in different applications, with more than 10000 citations in the Scopus database [24], so that it can be considered as a golden standard when a new hypothesis has to be tested. Moreover, the possibility of including channel gating stochasticity allowed us to realistically model channel noise which is particularly relevant in the auditory fibers, due to their small size [25, 26].

To account for the random gating of sodium and potassium channels, the ionic current densities *I*_*Na*_ = *g*_*Na*_(*V* − *E*_*Na*_) and *I*_*K*_ = *g*_*K*_(*V* − *E*_*K*_) were calculated using a channel-state-tracking algorithm [27, 28] where Markov chains [27, 29] modeled independent gating particles belonging to each ionic channel.

The magnitude of fluctuations in current densities (channel noise) depends on the number of ionic channels and, thus, for fixed channel densities (*ρ*_Na_=60 channels/*μ*m^2^, *ρ*_K_=18 channels/*μ*m^2^), on the area of the considered membrane patch. Specifically, channel noise is inversely proportional to the square root of the number of ionic channels in the membrane patch [21, 30]. Acoustic fibers are characterized by small Ranvier nodes, whose size may vary from 2.2 [25] to 15.7 *μ*m^2^ [26] and thus by high levels of intrinsic channel noise. In this work, three patch areas were considered: 2.2, 11.0, and 15.7 *μ*m^2^, corresponding to the maximum, the minimum, and an intermediate fiber size.

Besides Na, K, and leakage current densities,* I*_0_ represents here the background level of stimulation coming from the OHCs. This current density determines the firing rate of the neuron, i.e., the operating point on the IHC transfer function.

To simulate different states of pathologic neuron depolarization,* I*_0_ was set to a value close to the threshold: 6 *μ*A/cm^2^ and to suprathreshold values: 7 and 10 *μ*A/cm^2^ [23]. Conversely, physiological spontaneous firing of the auditory fiber was modeled by using a subthreshold bias current density* I*_0_ equal to 2 *μ*A/cm^2^. With respect to this physiological condition, the other conditions increased the background firing activity from 30 to 80%, as suggested by experimental recordings in animals with induced tinnitus [12, 13].

In this paper, for each patch area, four bias currents densities were used: 2, 6, 7, and 10 *μ*A/cm^2^. The first value was used to model a healthy acoustic fiber; the other ones modeled paroxysmal excitation underlying tinnitus.

The model was run in the C++ environment using the forward Euler integration method with time step 10 *μ*s.

In principle, the HH model extends its validity up to frequencies that short-circuit the membrane capacitance. According to [31], this occurs above the beta relaxation frequency of the cell membrane, at about 100 MHz. Moreover, the ionic channel modeling using Markov chains [32] is valid if the sampling time is much longer than the channel protein transition time (order of ps) [33]. The used time step of 10 *μ*s imposes a practical limitation of 50 kHz to the maximum frequencies that can be studied with the model. This is well below the theoretical frequency limitations of the model previously discussed.

For each studied condition, 300 independent runs of the model, 1 s in duration, were considered. The number of runs was approximately the number of afferent fibers contemporary stimulated by a single electrode of the cochlear prosthesis; this number was calculated by considering the size of the electrode (0.3 mm), the diameter of a IHC (≈10 *μ*m), and the number of auditory fibers (≈10) contacting a single IHC.

### 2.2. Stimulation

The exogenous stimulation was introduced in the model as an additional voltage over the membrane potential [34–36]. In terms of equivalent HH electric circuit, the electric stimulus was represented as a voltage generator in series with the membrane capacitor and the ionic conductances per unit area [37–40].

The applied electric stimulation was either a CW or a zero-mean GWN or a combination of both.

It should be noticed that the CW is a deterministic signal completely characterized by amplitude (A) and frequency (f), whereas the GWN, being a stochastic process, is described by its statistic moments, namely, average value, variance (*σ*_N_^2^), and autocorrelation function.

The GWN had zero-mean value, flat spectrum, and variance values: *σ*_N_^2^=3, 25, 100 mV^2^. The variance can be associated with the average power that the process dissipates on a 1 Ω resistance. The CW signal was chosen to have amplitude values: A=1.73, 5, 10 mV, equal to the standard deviations (*σ*_N_) of the considered GWN processes, where *σ*_N_ was taken as a measure of the amplitude of noise fluctuations. The CW frequencies were chosen to be equal to 25, 35, 50 kHz because they are above the upper perception threshold of human hearing (20 kHz). Due to the time step of 10 *μ*s chosen for the model solution, 50 kHz is the maximum frequency allowed for an input signal. For the same reason, even the GWN spectrum is practically limited to that upper frequency.

After separately studying the two kinds of stimulation, all combinations of the CW signals and the GWN were applied to the model to check possible cooperative effects.

### 2.3. Quantification of Firing Reduction

As already mentioned in Introduction, a pathologic acoustic fiber exhibits a spontaneous firing rate higher than that of a healthy neuron [12, 13]. The mean firing rate, i.e., the number of spikes per second, is due to the operating point fixed by the OHC and to the endogenous noise related to the number of ionic channels. To quantify the level of firing inhibition, and thus of tinnitus suppression, induced by the electric stimulation, it is necessary to introduce a sensitive technique.

In this work, the inactivation function (IA) was defined as follows:

(2) $$\begin{array}{c} IA=\frac{\#spike\left(\sigma_{N}=0;A=0;f=0;I_{0}=6,7,10\right)-\#spike\left(\sigma_{N}\neq 0;A\neq 0;f\neq 0;I_{0}=6,7,10\right)}{\#spike\left(\sigma_{N}=0;A=0;f=0;I_{0}=6,7,10\right)-\#spike\left(\sigma_{N}=0;A=0;f=0;I_{0}=2\right)}\times 100 \end{array}$$

where #*spike*(*σ*_*N*_=0;* A*=0;* f*=0;* I*_0_=6, 7,10) is the number of spikes per second of a pathologic neuron (*I*_0_*=*6, 7, 10 *μ*A/cm^2^) in the absence of exogenous electric stimulation (*σ*_*N*_=0 mV;* A*=0 mV;* f*=0 Hz); #*spike*(*σ*_*N*_ ≠ 0;* A*≠0;* f*≠0;* I*_0_=6, 7,10) is the number of spikes per second of a pathologic neuron during the exogenous electric stimulation (*σ*_*N*_ ≠ 0 mV;* A*≠0 mV;* f*≠0 Hz); #*spike*(*σ*_*N*_=0;* A*=0;* f*=0;* I*_0_=2) is the number of spikes per second of a healthy neuron (*I*_0_*=*2 *μ*A/cm^2^) in the absence of exogenous electric stimulation (*σ*_*N*_=0 mV;* A*=0 mV;* f*=0 Hz).

This quantity furnishes the percentage of firing reduction obtained using the stimulation in the pathologic neuron with respect to the difference, in terms of firing activity, between a pathologic and a physiologic neuron. The inactivation function will be 0% if the stimulation does not change the number of spikes of pathologic neuron and 100% if the neuron activity is turned back to the physiologic one. In this latter case, tinnitus is considered completely suppressed. Inactivation could be also higher than 100% if the firing activity is reduced below the physiologic condition or negative if the effect of electric stimulation is excitatory instead of inhibitory.

## 3. Results 

### 3.1. Spontaneous Firing

The used stochastic neuron model exhibits a firing activity, quantified by the mean firing rate (spikes per second), that increases with the bias current density* I*_0_ injected in the model, as shown in Table 1. Even in subthreshold conditions (see second column of Table 1) a not null firing rate is observed, due to the energy injected into the system by channel noise, that increases as the Ranvier node area becomes smaller (Table 1).

The neuron firing rate is due to the contemporary presence of channel noise and bias current density; the first one is determined by the typical sizes of the acoustic Ranvier nodes, the second one accounts for the operating point set by the OHC on the IHC transfer function, according to [9].

As shown in Table 1, for the same patch area, the three bias current densities, used to mimic the neuron with tinnitus (pathologic condition), increase the firing activity with respect to the physiologic condition, here modeled using the subthreshold bias current density* I*_0_=2 *μ*A/cm^2^. These increases range from 21% (*I*_0_=6 *μ*A/cm^2^) to 35% (*I*_0_=10 *μ*A/cm^2^), for the 2.2 *μ*m^2^ patch area, from 25% (*I*_0_=6 *μ*A/cm^2^) to 40% (*I*_0_=10 *μ*A/cm^2^), for the 11.0 *μ*m^2^ patch area, and from 35% (*I*_0_=6 *μ*A/cm^2^) to 57% (*I*_0_=10 *μ*A/cm^2^), for the 15.7 *μ*m^2^ patch area (Table 1). This shows that when channel noise decreases, in correspondence of larger patch areas, bias current densities assume a stronger influence on neuron firing.

The increased firing activity obtained by using the close to threshold and the suprathreshold current densities reported in Table 1 agrees with the experimental recordings on animals with induced tinnitus, reporting an increase from 35 to 83% [12, 13].

In the next sections, it will be examined the efficacy of different exogenous electric stimulations (see Section 2.2) in reducing the firing activity of pathologic neurons down to physiologic conditions.

### 3.2. Effect of Different Electric Stimulations

**Figure 1:**
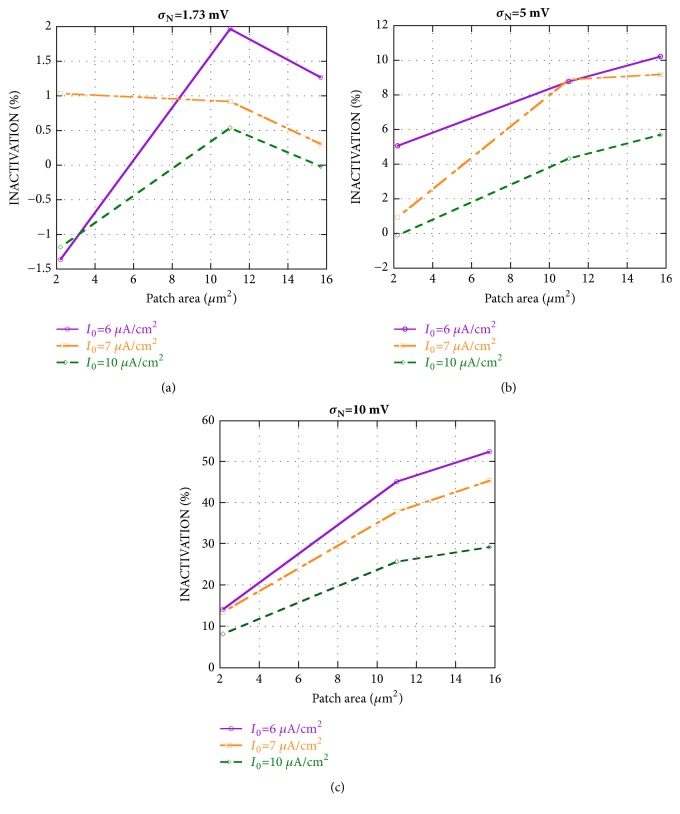
Inactivation versus patch area for different bias current densities* I*_0_. The exogenous stimulation is given by a Gaussian white noise GWN with different standard deviations: *σ*_N_=1.73 mV (panel (a)), *σ*_N_=5 mV (panel (b)), and *σ*_N_=10 mV (panel (c)).

The effects of a GWN on the mean firing rate of the neuron model, in each operating condition, have been quantified by the inactivation function IA, defined in Section 2.3, and summarized in Figure 1. For each pathologic condition, Figure 1 shows inactivation versus patch area for three standard deviations *σ*_N_ of noise fluctuations: 1.73 mV (panel (a)), 5 mV (panel (b)), and 10 mV (panel (c)).

For the lowest *σ*_N_ (Figure 1(a)), the inactivation does not exceed 2% and, in some cases, assumes negative values, indicating an increase of the mean firing frequency instead of a reduction. For *σ*_N_ of 5 mV (Figure 1(b)) it is possible to observe higher inactivation values that increase with the patch area and decrease with the bias current density, reaching a value of about 10% for patch size 15.7 *μ*m^2^ and bias current density 6 *μ*A/cm^2^. However, such values are too low to induce considerable tinnitus alleviation. Further increasing *σ*_N_ up to 10 mV (Figure 1(c)), the inactivation could become considerable, reaching 53% for the highest patch area and the smallest bias current density. However, the inactivation is just some percent points for the smallest patch area, where the endogenous channel noise dominates on the exogenous stimulation in determining the neuron firing rate.

Therefore, a standard deviation of 10 mV is necessary for the GWN to induce an inactivation from 26 to 53% in acoustic fibers whose Ranvier nodes are larger than 11 *μ*m^2^.

However, a broadband stimulation with a quite high power, related to the variance of noise fluctuations, may in principle induce unwanted acoustic perceptions coming from neighboring healthy hear cells.

Thus, it is worth evaluating the effect of using a stimulation with comparable amplitude of noise at a single frequency (CW) above 20 kHz, the upper perception limit of the human hearing. In fact, this stimulation cannot be directly interpreted as a sound by the human auditory system.

**Figure 2:**
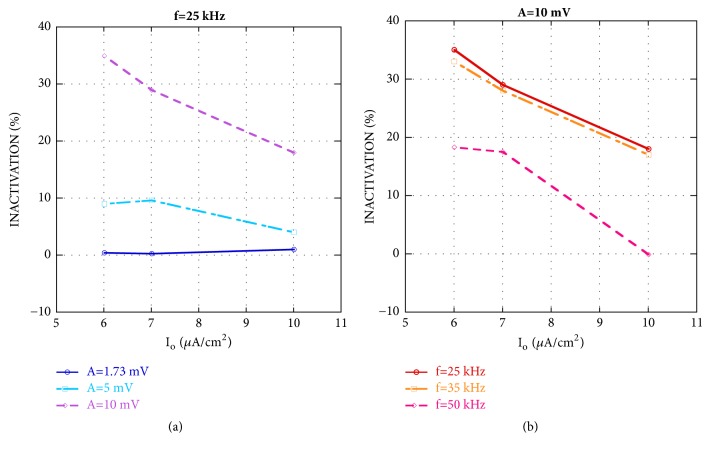
Inactivation versus bias current density* I*_0_ for a patch area equal to 15.7 *μ*m^2^. The exogenous stimulation is given by a CW at 25 kHz and amplitudes 1.73 mV, 5 mV, and 10 mV (panel (a)) or a CW of amplitude 10 mV and frequencies 25 kHz, 35 kHz, and 50 kHz (panel (b)).

Figure 2(a) shows the inactivation versus the bias current density for the larger patch area (best case) and an applied CW at 25 kHz and amplitude equal to 1.73, 5, or 10 mV. As discussed in Section 2.2, these amplitudes have been chosen to have the same standard deviation of the used GWNs.

Even in this case, the signal with 1.73 mV of amplitude is not efficient in inhibiting firing and that of 5 mV inactivates the neuron up to 10%. The effect becomes considerable for the 10 mV signal, when the inactivation is equal to 18% for* I*_0_=10 *μ*A/cm^2^ and reaches a maximum of 35% for* I*_0_=6 *μ*A/cm^2^. As already noticed for the GWN stimulation, the inactivation decreases with the bias current density, i.e., with the background firing activity of the pathologic neuron.

To evaluate the sensitivity to different stimulation frequencies, also 35 and 50 kHz CW signals have been considered.  Figure 2(b) shows the inactivation induced by 25 kHz, 35 kHz and 50 kHz CW signals with the amplitude set to 10 mV.

It is worth noticing that the CW is almost ineffective at 50 kHz, being the inactivation always less than 20%, whereas 25 kHz and 35 kHz signals behave in a similar way, with a slightly better performance of the 25 kHz CW. This evidences a frequency sensitivity of the neuron already observed also in a lower frequency range (50-500 Hz) [41, 42].

**Figure 3:**
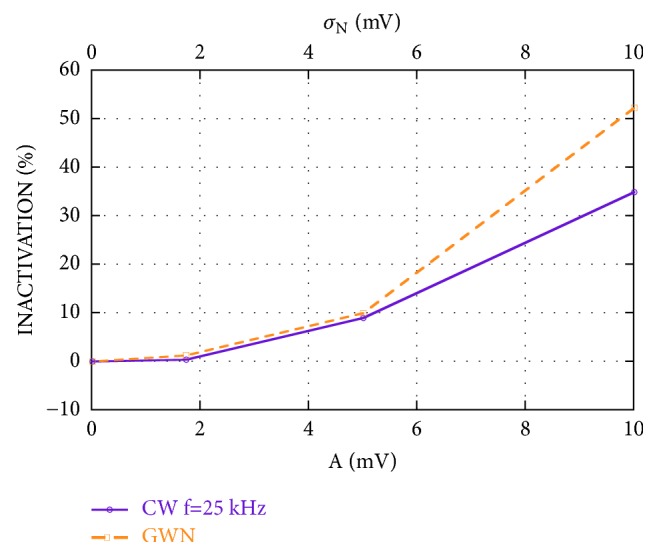
Inactivation induced by the CW stimulation versus the amplitude A of the CW at 25 kHz (purple solid line) and inactivation induced by the GWN stimulation versus its standard deviation *σ*_N_ (orange dashed line); A and *σ*_N_ assume the same values;* I*_0_=6 *μ*A/cm^2^; patch area=15.7 *μ*m^2^.

Results of simulations show that the GWN, having the standard deviation equal to the sinusoidal amplitude, is always more efficient than the 25 kHz CW in inducing firing reduction.  Figure 3 compares the inactivations induced by these two exogenous stimulations in the best case (*I*_0_=6 *μ*A/cm^2^; patch area=15.7 *μ*m^2^). Although the inactivation values are very similar when both the noise standard deviation (*σ*_N_) and the signal amplitude (A) are equal to 1.73 and 5 mV, for *σ*_N_=10 mV the inactivation induced by GWN is 52% versus 35% obtained by using the 25 kHz CW signal with the same amplitude. In fact, while the CW inactivation trend versus the amplitude (purple line in Figure 3) is accurately approximated (R=0.99976) by a quadratic curve with the second-order coefficient equal to 0.35, in the case of GWN (orange line in Figure 3), the quadratic function which best fits the inactivation trend (R=0.99964) has a second-order coefficient equal to 0.64.

To obtain 100 % inactivation, too high amplitude values for the CW signal would be necessary; conversely GWN has the disadvantage of having a spectrum segment in the auditory frequency band.

For these reasons, it would be useful to combine in a suitable way these two kinds of stimulation.

### 3.3. Effects of Combined Stimulation

The question arises on what happens if monochromatic and white stimulations are combined.

**Figure 4:**
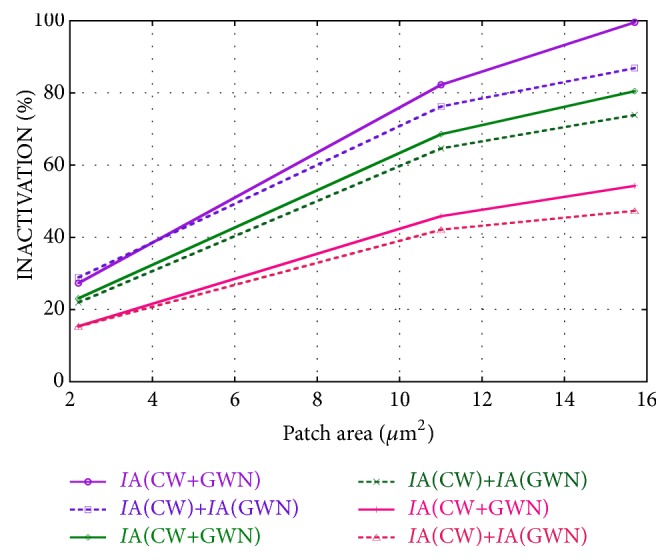
Inactivation IA versus patch area for* I*_0_=6 *μ*A/cm^2^ (purple lines),* I*_0_=7 *μ*A/cm^2^ (green lines), and* I*_0_=10 *μ*A/cm^2^ (magenta lines), obtained by combining the CW at 25 kHz 10 mV and the GWN, *σ*_N_=10 mV (solid lines), compared with the superposition of the inactivations induced by the two stimulations applied individually (dashed lines).

Results of the combined stimulation have been compared to the superposition of the effects induced by the two stimulations applied individually.  Figure 4 shows a comparison of inactivation obtained by combining the two kinds of stimulation IA(CW+GWN) with the sum of the inactivations obtained by using the two single stimulations IA(CW)+IA(GWN), in the best case: CW at 25 kHz with amplitude 10 mV, and GWN with *σ*_N_=10 mV.

As evident from Figure 4, except for the lowest patch area and* I*_0_=6 *μ*A/cm^2^, IA(CW+GWN) is always higher than IA(CW)+IA(GWN) and, for* I*_0_=6 *μ*A/cm^2^ and patch area 15.7 *μ*m^2^, it reaches 100%. This means that the firing rate of the stimulated neuron is reduced to physiologic conditions.

These results, due to the nonlinear neuronal behavior, show a cooperative effect of the applied signal and noise that can be usefully exploited in applications. So, a good stimulation solution could be a combination of CW and GWN to maximize tinnitus suppression while reducing possible side effects.

## 4. Discussion

Results of this work furnish a proof of concept that a suitable exogenous electrical stimulation, consisting of a high frequency (25-35 kHz) CW and/or Gaussian noise, can alleviate tinnitus through a mechanism of firing inhibition. This finding is coherent with studies on human volunteers, where the electrical stimulation was delivered to the cochlea [14–16], and suggests a possible interaction mechanism based on the reduction of the pathologic firing rate to the spontaneous activity of a healthy auditory fiber.

To simulate the single Ranvier node of an auditory fiber, a stochastic HH neuron model was used, since it is well characterized and considered as a reference model in the literature for a lot of different applications with more than 10000 citations in the Scopus database [24]. The authors themselves already used it to study neuronal encoding [37, 38, 42, 43] and to explain the analgesic effect of the Complex Neuroelectromagnetic Pulse [44] by means of a silencing mechanism [19].

A limitation of the used model is that, even if a temperature correction factor is used [45], it cannot work at the mammalian temperature of 37°C. In the HH model, a temperature increase causes the threshold current density to shift towards higher values, and the firing rate to change depending on the patch size [45]. So, different operating conditions, in terms of bias current densities, would mimic healthy and pathologic neuronal activities. Similar mechanisms of relative firing reduction are expected to occur for a suitable combination of signal and noise since the model anyway presents two attraction basins for firing ad resting states and the exogenous stimulation can push the system from one state to the other. However, since the temperature adjustment in neuronal models is still an open question, here it was preferred to use the well-assessed reference temperature for the HH model.

Due to the generality of the used model and the high number of degrees of freedom, a complete evaluation of the uncertainty budget is not practicable but, besides the temperature, the other main variables that may influence results are examined in the following.

An aspect that could contribute to the uncertainty of results is that, for frequencies above 10 kHz, the membrane capacitance per unit area (C_m_) is not constant, differently from what was assumed in our model. In fact, the permittivity of the cell membrane decreases with frequency due to the relaxation of the alpha polarization phenomenon [46]. Nevertheless, our simplification is largely acceptable since the frequency dependence of C_m_ was shown to have a negligible effect on the stimulation threshold of a HH model (median = 1.4%) [47].

Other model parameters that induce a great variability of results are the bias current density* I*_0_ and the patch area. When applying a combination of the CW (f=25 kHz, A=10 mV) and the GWN (*σ*_N_=10 mV) to the neuronal patch of 15.7 *μ*m^2^, the inactivation ranges from 54% (*I*_0_=10 *μ*A/cm^2^) to 100% (*I*_0_=6 *μ*A/cm^2^). Conversely, for* I*_0_=6 *μ*A/cm^2^, the inactivation passes from 28% to 100% if the patch size increases from 2.2 to 15.7 *μ*m^2^. Such variations could explain the great variability of results on human volunteers [16] that could be attributed to the individual variability of auditory fiber size (patch area in the model) and tinnitus severity (bias current density in the model).

This study suggests a plausible mechanism of tinnitus suppression using exogenous electrical excitation and is a first step towards the characterization of kind and parameters of stimulation that maximize the efficacy while reducing possible short-term or long-term side effects, such as unwanted sound perception or adaptation.

To control side effects, charge-balanced signals should be used and the induced currents should not exceed typical currents used in cochlear prostheses. A recent dosimetric study [48] revealed that a typical cochlear implant delivered, at the location of the afferent fibers of the auditory nerve, a peak voltage of several tens of mVs, higher than the signal amplitudes used in this work (≤ 10 mV). This suggests that the stimulation signals used in this work are plausible to be released from cochlear implants without severe side effects, even though it will be necessary to conduct a careful risk analysis to assess the safety of the proposed technique.

## 5. Conclusions

A stochastic HH neuron model was used to evaluate the efficacy of different electric stimulation strategies in tinnitus suppression. The used stimulations were CW signals at different frequencies in the range of tens of kHz and GWN.

Results of simulations show that both a CW and a white noise, applied individually to the neuron model, may induce a firing inhibition. The inactivation level is shown to depend on many parameters, such as patch area, bias current density, CW frequency and amplitude, and noise standard deviation. The more the background activity is low (larger patch size and lower bias currents), the more the inactivation is high. Considerable inactivation values are obtained by using either CW at 25 or 35 kHz or GWN with 10 mV of standard deviation, but GWN is shown to be more efficient than CW (IA=53% versus IA=35% in the best condition) for a comparable amplitude of fluctuations.

Moreover, the inactivation induced by a combination of signal and noise is almost always higher than the sum of the inactivations induced by the two stimulations applied individually and it reaches 100% for the lowest* I*_0_ and the highest patch area.

These results are a proof of concept that signal and noise act on the neuron in a cooperative way and could be suitably delivered in combination through cochlear prosthesis to alleviate tinnitus while reducing possible side effects due to a broadband stimulation.

Future works will concern the validation of the presented results on a mammalian neuronal model at 37°C, such as the Spatially Extended Nonlinear Node (SENN) [49] and the McIntyre-Richardson-Grill (MRG) [50] models and the identification of a colored stimulating noise suitably filtered considering the typical frequency selectivity of the used model.

## Figures and Tables

**Table 1:** Mean firing rate (spikes/s) exhibited by the neuron model for different bias current densities *I*_*0*_ and patch areas in the absence of external electric stimulation.

	***Sub-threshold (physiologic)***	***Close to threshold*** *** (pathologic)***	***Supra-*** ***threshold *** ***(pathologic)***	
*Patch area (μm* ^*2*^)	*I*_*0*_ *=2 μA/cm* ^*2*^	*I*_*0*_ *=6 μA/cm* ^*2*^	*I*_*0*_ *=7 μA/cm* ^*2*^	*I*_*0*_ *=10 μA/cm* ^*2*^

2.2	53.7	64.9	66.8	72.2

11.0	44.5	57.8	60.7	67.2

15.7	42.1	56.5	59.3	66.2
